# Brown trout in Oder estuary tributaries: genetic structure, stocking, and admixture

**DOI:** 10.1007/s13353-024-00890-z

**Published:** 2024-07-03

**Authors:** Rafał Bernaś, Anna Wąs-Barcz, Waldemar Święcki, Piotr Dębowski, Grzegorz Radtke, Adam Tański, Agata Korzelecka-Orkisz, Krzysztof Formicki

**Affiliations:** 1 Department of Migratory Fish, National Inland Fisheries Research Institute, Rutki 49, 83‑330 Żukowo, Poland; 2 Department of Fisheries Resources, National Marine Fisheries Research Institute, Kołłątaja 1, 81-332 Gdynia, Poland; 3 Department of Logistics and Monitoring, National Marine Fisheries Research Institute, Kołłątaja 1, 81-332 Gdynia, Poland; 4 Department of Hydrobiology, Ichthyology and Biotechnology of Reproduction, West Pomeranian University of Technology in Szczecin, Kazimierza Królewicza 4, 71-550 Szczecin, Poland

**Keywords:** *Salmo trutta*, Sea trout, Resident brown trout, Stock composition, Parentage analysis, Biodiversity conservation

## Abstract

Several rivers that are tributaries of the Oder estuary are inhabited by *Salmo trutta* L, the most important of which are Ina, Gowienica, and Wołczenica. Both forms of the species, sea trout and resident brown trout, are present. All rivers are traditionally stocked with either sea trout from the neighboring Pomeranian river Rega basin or resident brown trout from various locations. To examine populations in these rivers in terms of genetic structure, genetic diversity, and origin, they were analyzed using 13 microsatellite loci. Relatedness was also assessed for fish stocked in the same year. The obtained genotypes were compared with breeding stocks used for stocking in Poland. The analyses revealed a significant genetic distance between adult individuals from Ina and Rega Rivers and fish caught during electrofishing. Strong kinship relationships were identified in the sampled areas, with high proportions of fish originating from stocking and their dominance in numbers over wild juveniles, primarily in smaller tributaries. Additionally, clear separation in the origin of stocked individuals was observed. Adult trout from Ina and Rega are genetically closer to northern brown trout lineages, providing crucial information for the management and biodiversity conservation of Polish *Salmo trutta* populations.

**Supplementary Information:**

The online version contains supplementary material available at 10.1007/s13353-024-00890-z.

## Introduction

Stocking is among the most common methods for increasing fish populations (Cowx 1994; Arlinghaus et al. 2002), but it remains controversial (Naish et al. 2007). On one hand, it can effectively support weak populations or serve as a source for targeted fishing, but on the other hand, it can cause more harm than good, especially genetic and epigenetic issues (e.g., Linløkken et al. 2014; Bernaś et al. 2020; Berrebi et al. 2021). Whether stocking should be employed for protecting natural populations is at least debatable (Fujitani et al. 2016), but it is undoubtedly a widely used practice, especially in areas with intensive fishing exploitation.

One of the fish species commonly subjected to stocking worldwide is the brown trout, *Salmo trutta* L. This paleoarctic species occurs naturally in Europe from the White Sea to North Africa and from Great Britain to Western Asia (Klemetsen et al. 2003). It is a highly polymorphic bony fish with several life strategies (Jonsson and Jonsson 2017). Anadromous brown trout, also known as sea trout, migrate from the rivers or streams of their origin to the sea, where they feed until reaching sexual maturity, before returning to the rivers where they were born for spawning. In contrast, resident brown trout spend their entire lives in rivers or streams and often spawn in smaller tributaries further upstream in the same river area (Elliott 1994). Stocking practices related to this species primarily concern Europe, where stocking with hatchery-reared brown trout for over a century has aimed to expand natural populations (Hansen and Loeschcke 1994; Berrebi et al. 2021). Currently, the main goal of stocking this species is to support recreational fishing, and in the case of anadromous forms, coastal fisheries (Naish et al. 2007; Wąs-Barcz and Bernaś 2023). Sea trout stocking is particularly prevalent in countries within the Baltic Sea watershed (ICES 2023).

Currently, there are ~ 600 sea trout populations in the Baltic Sea watershed (ICES 2023). In Poland, there are ~ 25 sea trout rivers in which natural spawning occurs, mainly in the northern part of Poland (Pomerania), but also in the Vistula and Oder River basins. Historically, this ecotype had a much larger distribution range, with the most extensive spawning areas located in the Carpathian tributaries of the Vistula. However, these areas have been cut off due to the construction of numerous migration barriers (Dębowski 2018). The resident form of brown trout has a significantly wider distribution range, mostly coinciding with the historical distribution of the anadromous form, expanded by new locations resulting from stocking efforts (Bernaś and Wąs-Barcz 2020).

The genetic population structure of brown trout in the Baltic Sea watershed is reasonably well understood. In the case of Poland and the southern Baltic Sea area, the genetics of local populations are also quite well-characterized (e.g., Poćwierz-Kotus et al. 2014; Schmidt et al. 2017; Petereit et al. 2018; Burimski et al. 2023). Currently, the level of variability both within and between Pomeranian sea trout populations usually falls within the range of *F*_ST_ 0.01 − 0.03, which is typical for other adjacent Baltic sea trout rivers (e.g., Lehtonen et al. 2009; Östergren and Nilsson 2012; Koljonen et al. 2014; Petereit et al. 2018). Overall, the best-studied populations from Poland are those from the Vistula, Słupia, Parsęta, and Rega Rivers, which have been extensively examined, including mitochondrial DNA analysis (e.g., Bernatchez 2001; Wąs and Wenne 2003; Bernaś et al. 2014, 2021; Wenne et al. 2016). However, populations from the lower Oder River tributaries have not been studied to date.

The main goal of this research was to determine the level of genetic diversity and the genetic structure of brown trout populations in the Oder estuary area. This included describing the impact of stocking with different forms of the species in the basins and assessing their interrelationships, also considering their genetic position relative to other Polish brown trout populations and broodstocks. Achieving these goals will also serve to test the hypothesis that individuals from stocked areas can dominate over wild ones during the nursery season in the areas where they are released.

## Material and methods

### Research area

**Fig. 1:**
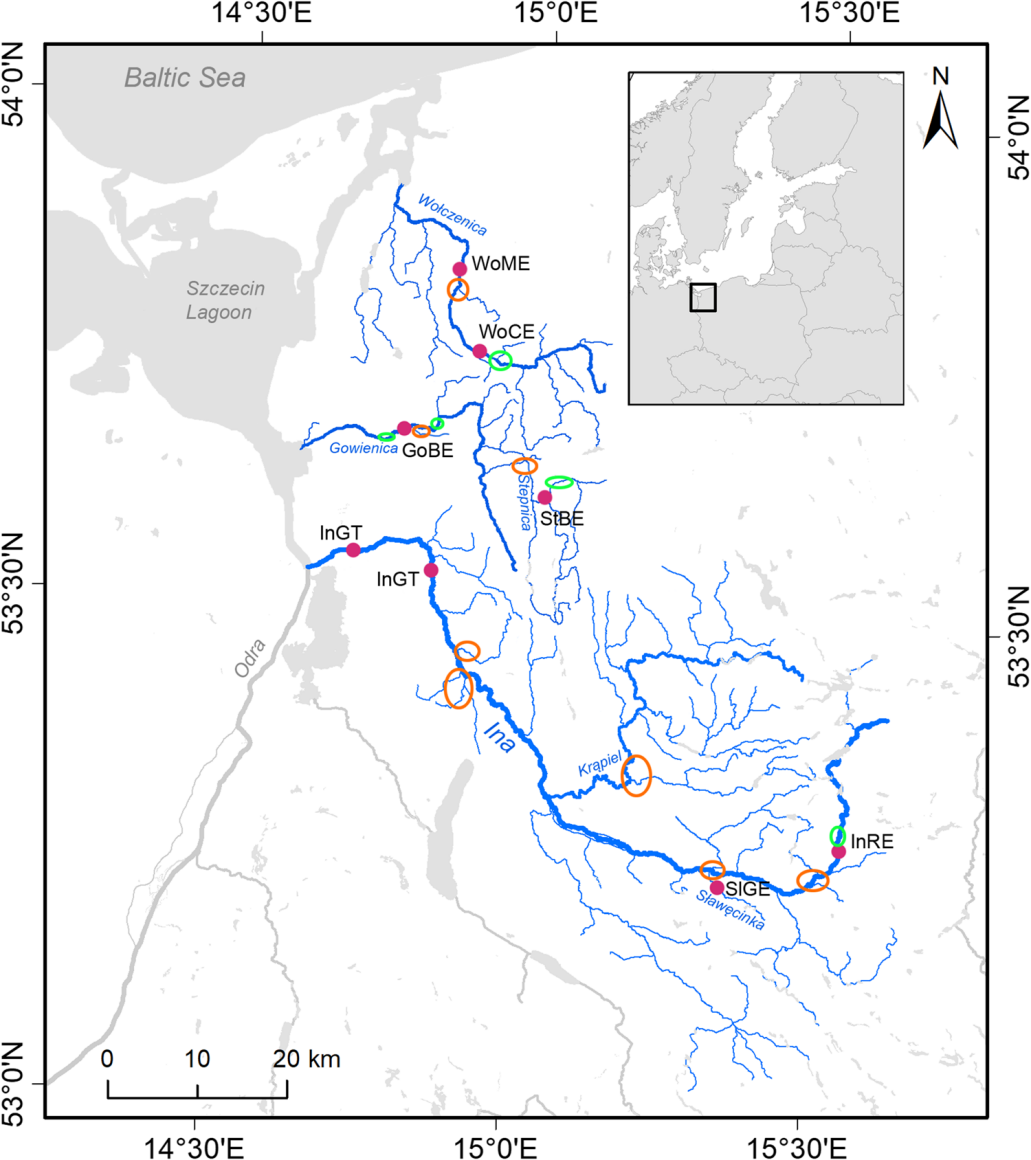
Map showing the locations of sampled sites in the Ina, Wołczenica, and Gowienica basins (pink dots), and areas stocked with resident brown trout progeny (green ellipses) and sea trout progeny (orange ellipses)

The rivers under study are right-bank tributaries of the Oder estuary. The largest among them, the Ina River, flows into the Oder between the outflow from Lake Dąbie and the southern part of the Szczecin Lagoon. Slightly further north is the mouth of the Gowienica River, which flows into the bay of the Szczecin Lagoon near the Stepnica village. In turn, the Wołczenica River flows into the Dziwna Strait, connecting the waters of the Szczecin Lagoon with the Baltic Sea (Robakiewicz 1993) (Fig. 1).

The Ina River is 128 km long, and its drainage basin covers an area of 2151 km^2^. Its average gradients in the upper and lower courses are 4‰ and 0.2‰, respectively. The average flow at the estuary is 10 m^3^/s. Currently, thanks to a fishway system, the river is potentially open for migration along almost its entire length up to the mill of Rybaki (Fig. 1).

The Gowienica River is 51 km long with a drainage basin of 368 km^2^. It is fully accessible for fish migration up to the old mill in Babigoszcz (19.5 km) (Jankowski et al. 2017). The Wołczenica River is 52 km long with a drainage basin of 462 km^2^. It is accessible for fish migration up to the small hydroelectric facility in Derkacz (25.5 km) (Tański et al. 2018). In a recent fish fauna study on the Ina River, 33 species of fish and lampreys were identified, with migratory trout, perch, and bullhead dominating (Keszka et al. 2013).

### Management

All three rivers have populations of brown trout. The resident form is most numerous in the upper reaches of the studied rivers, often isolated by hydrotechnical structures (Fig. 1). All studied rivers are under the management of the Polish Angling Association District in Szczecin and are stocked with migratory trout (from the Rega River population) and resident brown trout (often from the Folusz Breeding Center) of non-local genetic lines. Stocking of the Ina River with Rega sea trout has been practiced by the Polish Angling Association Region Szczecin since the 1960s. Between 250,000 and 500,000 migratory trout fry and around 40,000 resident brown trout alevins are introduced into the Ina River annually. Gowienica and Wołczenica are stocked annually with ~ 50,000 migratory trout fry and 8000 resident brown trout alevins (HELCOM 2011; ICES 2023).

### Sample collection and DNA extraction

First, samples were collected from stocking material from anadromous and resident forms, before they were released into the Ina, Wołczenica, and Gowienica in the spring of 2021 as fry (Table 1). Stocking material for sea trout was from spawners collected in the neighboring Rega River. Resident brown trout were from the Folusz hatchery located in southern Poland (Bernaś and Wąs-Barcz 2020).

**Table 1:** Details of brown trout used for stocking and collected in the Ina, Gowienica, Wołczenica, and Rega Rivers in 2021–2022

Abbreviation	River/released to	Date	GPS/origin	Ecotype	*N*	Age
InAS	Ina	April 2021	Stocked	Anadromous	32	Fry
InRS	Ina	April 2021	Stocked	Resident	31	Fry
GoAS	Gowienica	April 2021	Stocked	Anadromous	32	Fry
GoRS	Gowienica	April 2021	Stocked	Resident	30	Fry
WoAS	Wołczenica	April 2021	Stocked	Anadromous	32	Fry
WoRS	Wołczenica	April 2021	Stocked	Resident	30	Fry
InRE	Ina	27.07.2021	53.289255, 15.546678	Facultatively anadromous	32	0 + to 1 +
GoBE	Gowienica	28.07.2021	53.680190, 14.811697	Facultatively anadromous	32	0 + to 2 +
WoCE	Wołczenica	28.07.2021	53.759733, 14.906575	Facultatively anadromous	32	0 + to 1 +
WoME	Wołczenica	28.07.2021	53.804672, 14.848073	Facultatively anadromous	18	0 + to 1 +
SlGE	Sławęcinka	07.09.2021	53.249111, 15.332973	Facultatively anadromous	32	0 + to 1 +
StBE	Stepnica	28.07.2021	53.644214, 14.935033	Facultatively anadromous	32	0 + to 2 +
InGT	Ina	10.2022	53.551865, 14.838841	Anadromous	20	Spawners
ReTE	Rega	11.2021	54.064793, 15.269579	Anadromous	40	Spawners

From left: sample abbreviation, river of origin or release, sampling date, GPS location for sampled from rivers, ecological form, *N*, number of specimens, age of sampled fish

Field sampling began in summer 2021 when 178 brown trout were collected from six locations. These were fish of various ages between 0 and 2 + years. Additionally, 20 anadromous adults were collected from the lower part of the Ina River between the river mouth and Goleniów during electrofishing for telemetry survey in autumn 2022 (Fig. 1, Table 1). The mean size of anadromous spawners was 63.8 cm (52 − 80 cm). To facilitate interpretation and verify actual relationships, the last group added to the analysis was a sample of 40 adult sea trout from the Rega River, caught during spawning migration in Trzebiatów during autumn 2021.

Fin clip samples (approximately 2 − 5 mm^2^) were collected from 425 individuals. Genomic DNA was extracted from fin tissue preserved in 96% ethanol using a Genomic Mini Kit (A&A Biotechnology, Gdynia, Poland) and diluted to a concentration of 30 − 100 ng/μl. Sampling details are presented in Table 1. Regarding sample abbreviations, the first two letters indicate the name of the river, the third the locality or origin, and the fourth additional details (E, electrofishing; T, telemetry; S, stocked).

### Microsatellite analysis

For microsatellite analysis, a set of 13 fluorescently labeled polymorphic microsatellite loci (*OneU9*, *Strutta58P*, *Ssosl438*, *Ssosl311*, *Str15INRA*, *Str543INRA*, *Str60INRA*, *Str73INRA*, *Ssosl417*, *Str85INRA*, *Ssa85*, *Bs131*, and *Ssa407*) were amplified by single multiplex PCR using a Qiagen Multiplex PCR Kit (Qiagen, Hilden, Germany). Multiplex PCR (7 μl) contained ~ 100 ng of template DNA, multiplex PCR master mix, and 0.2 − 0.6 μM of each primer. Amplifications were carried out in a TProfessional Basic Gradient thermal cycler (Biometra) with an initial denaturation step at 95 °C for 5 min followed by 38 cycles of denaturation at 94 °C for 30 s, annealing at 55 °C for 90 s, and extension at 72 °C for 60 s. Reactions were terminated after 30 min, and a final extension was performed at 60 °C. PCR products were genotyped via single capillary electrophoresis on an ABI Prism 3130xl genetic analyzer (Applied Biosystems) along with GeneScan 600LIZ size standards (Applied Biosystems). DNA fragments were estimated using a Peak Scanner v1.2 (Applied Biosystems).

### Statistical analysis

Observed and expected heterozygosity and the mean number of alleles (total number of alleles at all loci divided by the number of loci) were calculated using Arlequin 3.5.2.2 (Excoffier and Lischer 2010). Population-specific *F*_IS_, pairwise-weighted *F*_ST_ values over all loci based on the number of different alleles, and Nei’s genetic distances were also determined with this software. Departures from the Hardy–Weinberg equilibrium (HWE) were detected with Chi-square tests in GenAlex 6.5 (Peakall and Smouse 2012). HPRARE was used to calculate allelic richness (which allows comparison of allele numbers without the bias associated with different sample sizes) and the richness of private alleles (alleles limited in a single population) (Kalinowski 2005). Overall, the *F*-statistic (*F*_ST_, *F*_IT_, *F*_IS_) was estimated by analyzing molecular variance (AMOVA) implemented in Arlequin 3.5.2.2.

STRUCTURE 2.3.4 was applied to detect genetic structure and gene flow (Pritchard et al. 2000). The Evanno method (ΔK) (Evanno et al. 2005) was chosen to infer the highest number of clusters (*K*) based on the rate of change in log probability among consecutive *K* values. Five iterations of each* K* were performed with 200,000 burn-ins and 200,000 Markov chain Monte Carlo (MCMC) repetitions. Clumpak was then used to identify the optimal alignment of inferred clusters across different values of *K* (Kopelman et al. 2015).

In order to perform family structure parentage analysis, Colony 2.0.6.6. (Jones and Wang 2010) was employed for each electrofished sample together with corresponding samples from anadromous and resident forms released in the area of the given location. This analysis was also performed for adult sea trout from the Ina and Rega Rivers, which were caught during their spawning migration in 2021 and 2022. This was to determine the actual population parameters in a random sample, the most representative among the population in the Ina. We applied non-default COLONY job settings including typing error rate 0.001, mating system I with male and female polygamy, mating system II with inbreeding, run length medium, and analysis method FL, with defaults for other settings. The main goal was full-sib and half-sib dyad detection, determination of the number of families, and estimation of the effective population size N*e*.

Additionally, to better understand and present genetic diversity, the obtained genotypes were compared with brown trout stocks used for stocking in Poland (Bernaś and Wąs-Barcz 2020). A neighbor-joining (NJ) phylogenetic tree was constructed based on pairwise Nei’s standard genetic distance without sample size correction (*D*_ST_) with 10,000 bootstrap replications using PopTree2 software (Takezaki et al. 2010).

## Results

### Genetic polymorphism and diversity

The mean number of alleles in individuals from investigated stocks ranged between 8.07 and 10.76 (Table 2). The highest number was found in stocks from Gowienica (GoBE) and Stepnica (StBE), although overall the differences were minimal. Heterozygosity observed in all cases was higher than expected, except for the batch of sea trout released into Wołczenica (WoAS). Regarding allelic richness, the highest values were observed in Gowienica (GoBE) and among Stepnica (StBE). Deviations from HWE occurred only for single loci. Population-specific *F*_IS_ values were significant (*p* < 0.05) with positive values for fish from Gowienica River (GoBE). In the case of anadromous fish stocked to the Wołczenica River (WoAS), the value of the inbreeding coefficient was negative, though this result was not statistically significant.

**Table 2:** Statistics of brown trout used for stocking and collected in the Ina, Gowienica, Wołczenica, and Rega Rivers in 2021–2022

Abbreviation	*N*	*M*_NA_	*H*_O_	*H*_E_	*A*_R_	*P*_AR_	*D*_HWE_	*F*_IS_
InAS	32	9.23	0.68	0.71	7.36	0.17	1	0.041
InRS	31	10.3	0.71	0.74	7.88	0.2	1	0.039
GoAS	32	8.07	0.66	0.69	6.43	0.04	1	0.049
GoRS	30	9.15	0.71	0.74	7.52	0.14	0	0.045
WoAS	32	8.07	0.72	0.7	6.38	0.07	1	− 0.028
WoRS	30	9.69	0.7	0.73	7.71	0.09	0	0.044
InRE	32	9.92	0.69	0.72	7.65	0.15	1	0.044
GoBE	32	10.76	0.63	0.72	8.15	0.2	1	**0.129**
WoCE	32	9.46	0.71	0.74	7.68	0.09	1	0.040
WoME	18	8.07	0.7	0.72	7.52	0.24	1	0.032
SlGE	32	8.23	0.7	0.71	6.79	0.07	2	0.018
StBE	32	10.46	0.73	0.75	8.08	0.21	0	0.021
InGT	20	7.61	0.73	0.68	7.06	0.24	2	0.023
ReTE	40	9.23	0.66	0.68	7.06	0.29	1	0.027

Significant values were bolded (*p* < 0.05)

*N*, number of fish; *M*_*NA*_, mean allele number in the population; *H*_*O*_, observed heterozygosity; *H*_*E*_, expected heterozygosity; *A*_*R*_, allelic richness; *P*_*AR*_, private allele richness; *D*_*HWE*_, number of loci with deviations from HWE; *F*_*IS*_, stock-specific inbreeding coefficient

Overall *F*_ST_ value obtained by AMOVA for all pairs of loci was 0.046 and was significant. The highest percentage of variation was detected within individuals at 92%. Overall, *F*_IS_ and *F*_IT_ reached 0.03 and 0.07, respectively, and were significant (*p* < 0.05).

The highest *F*_ST_ values for pairwise differences were between adult spawners from the lower Ina River (InGT) and Rega River (ReTE) and all other studied locations (0.127 < *F*_ST_ < 0.173, Table 3). The level of this differentiation was moderate sensu (Hartl and Clark 2006) and was lowest for sea trout from Sławęcinka (SlGE). The lowest and non-significant pairwise differences were detected for samples collected in the upper Ina (InRE) and Stepnica (StBE) (*F*_ST_ = 0.005). Other insignificant cases concern comparisons with individual batches of fish released as part of stocking activities and for spawners from Ina and Rega (InGT) vs. (ReTE) (Table 3).

**Table 3:** Pairwise *F*_ST_ genetic diversity indices of brown trout samples collected in the Ina, Gowienica, Wołczenica, and Rega Rivers, and fish used for stocking in 2021

Pop	InAS	InRS	GoAS	GoRS	WoAS	WoRS	InRE	GoBE	WoCE	WoME	SlGE	StBE	InGT
InRS	0.037												
GoAS	0.024	0.039											
GoRS	0.048	***0***	0.042										
WoAS	0.02	0.038	***0***	0.043									
WoRS	0.053	***0***	0.048	***0***	0.048								
InRE	0.018	***0.005***	0.027	0.006	0.032	0.013							
GoBE	0.028	0.02	0.028	0.02	0.037	0.023	0.008						
WoCE	0.054	***0.003***	0.045	***0.002***	0.047	***0.004***	0.016	0.022					
WoME	0.022	0.035	***0.009***	0.039	0.013	0.041	0.024	0.012	0.043				
SlGE	0.01	0.031	0.025	0.034	0.026	0.04	0.013	0.015	0.041	0.023			
StBE	0.033	***0.004***	0.04	0.008	0.04	0.009	***0.005***	0.02	0.019	0.035	0.026		
InGT	0.146	0.138	0.159	0.132	0.158	0.138	0.141	0.128	0.129	0.149	0.127	0.144	
ReTE	0.157	0.152	0.173	0.148	0.17	0.156	0.154	0.148	0.148	0.164	0.143	0.156	***0***

*F*_ST_ values for pairwise comparisons that were not significant (*p* > 0.05) are in bold italics

### Genetic structure and admixture

**Fig. 2:**
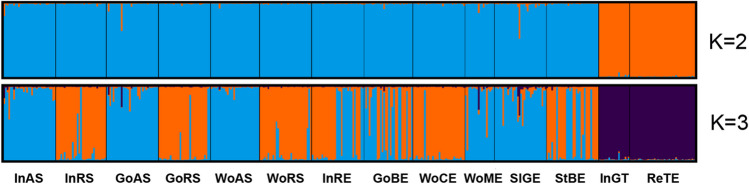
Clustering of brown trout from analyzed stocks/populations with putative *K* = 2 (upper bar) and *K* = 3 (lower bar). Each individual is represented by a column divided into *K* shades with each shade representing a cluster

Bayesian estimation of genetic structure and memberships indicated that the maximum value of Δ*K* was for *K* = 2 (Δ*K* = 532.88) followed by *K* = 3 (Δ*K* = 220.40; Supplementary Fig. S1, Fig. 2). Regarding the separation logic for *K* = 2, the first cluster is the genotypes characteristic for all studied groups except adult sea trout from Ina and Rega Rivers. In the case of the *K* = 3 scenario, the first cluster (blue) are the genotypes of the offspring of anadromous fish from Rega, the second cluster (orange) are the genotypes of the offspring of the resident line from Folusz, and the third cluster (purple) are genotypes characteristic of adult sea trout from Ina and Rega.

**Fig. 3:**
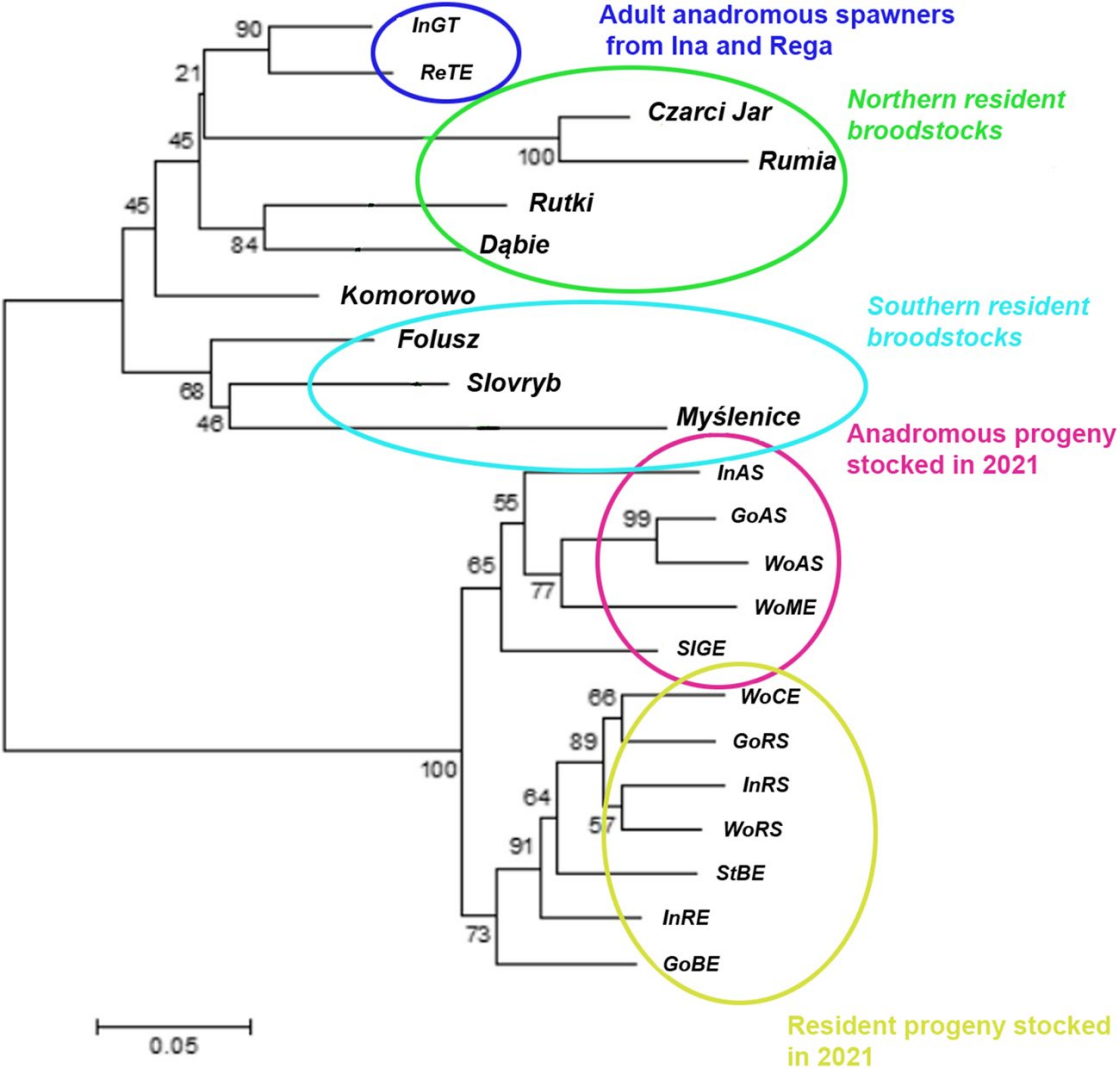
Bootstrapped NJ phylogenetic tree for all analyzed individuals along with stocks of resident brown trout used for stocking in Poland (Bernaś and Wąs-Barcz 2020)

Phylogenetic tree analysis showed that anadromous adults from Ina and Rega groups corresponded with stocks of resident brown trout from northern breeding lines, not southern lines. Separation from the stocking material from 2021 was also very clear. In stocked fish, there was a clear division into a group representing resident and anadromous genotypes (Fig. 3).

### Familial structure and sibling detection

**Fig. 4:**
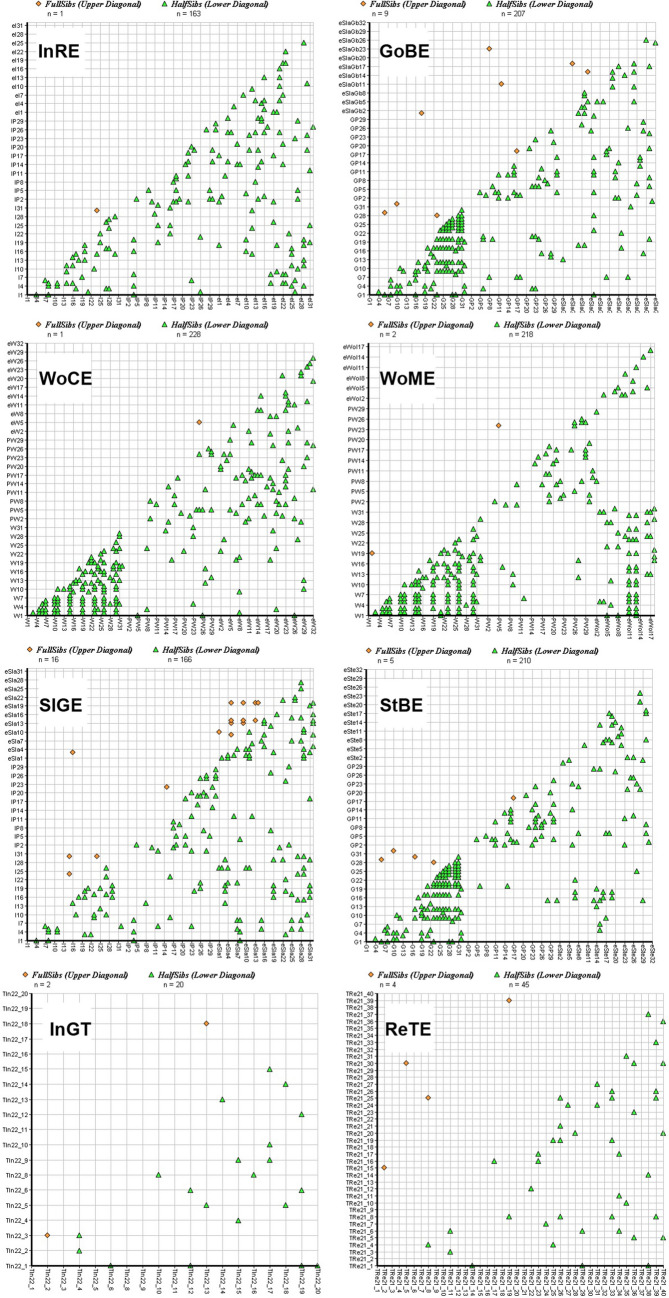
Relatedness among analyzed brown trout collected in Ina, Rega, Gowienica, and Wołczenica Rivers and used for stocking in 2021

Analysis of familial structure and effective population size calculated by COLONY showed that the proportions of unrelated individuals within samples varied from only 32.09% (WoME) to 83.98% (InRE) (Table 4, Fig. 4). This is unsurprising considering that juveniles and spawning locations were sampled, which significantly increases the chances of detecting related individuals. However, the purpose of this analysis was primarily to detect stocking individuals. The largest number of such individuals among full siblings was detected in Gowienica and Stepnica (Table 4). The lowest values of related individuals occurred in the sample of adult trout from Ina that were caught during telemetry research in 2021 and 2022.

**Table 4:** Results of sibship assignment employed by COLONY

Stock	Full-sib families	Unrelated dyads	Full-sib dyads	Half-sib dyads	*Ne* (CI95 L-U)
InRE	94	860 (83.98%)	1 (L1)	163	108 (81 − 147)
GoBE	85	808 (78.92%)	9 (L2, SR2, SA1, AS4)	207	78 (56 − 110)
WoCE	93	798 (77.90%)	1 (SR1)	225	76 (54 − 106)
WoME	78	104 (32.09%)	2 (AS2)	218	57 (40 − 86)
SlGE	86	842 (82.22%)	16 (L12, SA2, AS2)	166	90 (66 − 125)
StBE	89	809 (79.00%)	5 (AS5)	210	79 (57 − 111)
InGT	17	339 (97.63%)	2	20	76 (50 − 123)
ReTE	36	1551 (96.9%)	4	45	60 (37 − 94)

Full-sib and half-sib dyads are displayed as both absolute values and relative frequencies (brackets). Full-sib families concern all detections, including single detections. The last column is the effective population size. Full-sib detections also show the number and origin of the detected relationships

*L*, local fish; *SR*, stocked resident; *SA*, stocked anadromous; *AS*, among stocked

## Discussion

During our research, we determined the genetic structure in the examined locations, with a clear distinction between resident brown trout of non-local origin. This is not a situation that should be considered appropriate because in typical natural populations, gene flow between ecotypes of brown trout is maintained, and they usually genetically constitute a single population, at least within the range of the anadromous form (Charles et al. 2005). This is the case, for example, in the adjacent Parsęta River (Bernaś et al. 2021). Additionally, in most locations, there was a distinct mixture of gene pools from fish of different origins. These include genotypes typical for juveniles stocked as Rega sea trout (blue cluster), resident trout of non-local origin (orange), and most likely wild progeny of local fish (purple). This observation is supported by the fact that this cluster corresponds to adult sea trout from Ina and Rega. A notable example of this phenomenon is the high and significant population inbreeding coefficient for the sample from Gowienica. In this case, the indicator does not inform about relatedness, but rather suggests the existence of a strong substructure (homozygotes excess). This serves as a model example of the Wahlund effect (Ridley 2004). A slightly insignificant opposite case occurred with the stocked anadromous fish from Wołczenica, where we observed negative values of the inbreeding coefficient. This suggests the occurrence of excess heterozygotes. This situation may be the result of mixing different gene pools during artificial spawning.

It is particularly interesting that adult trout from the Ina River deviate significantly from the other examined locations and are very similar to adult trout from the Rega River. However, it is certain that the Ina River basin has been stocked with migratory trout from the Rega River since at least the 1960s, so the lack of distinctiveness in the Ina population compared to the Rega population is unsurprising (NIFRI data).

The fact that there is a clear mixture of genotypes in all examined locations is, of course, influenced by stocking, as confirmed by relatedness analyses, where many full-sibling detections concern fish from the analyzed stocking groups. This situation does not occur among migratory adult trout from the Ina and Rega Rivers. The results also indicate that, from the perspective of the population, resident/migratory trout from Wołczenica and Gowienica are more influenced by stocking than the population from the Ina River. This is logical, considering the size of the basins and the population sizes. The analysis also shows how clearly stocked resident brown trout are separated from stocked migratory sea trout which, of course, results not from differences between ecotypes but from different origins (southern stock). At the same time, the obtained results of relatedness analyses indicate that the hypothesis about the possible dominance of stocked individuals in terms of their numbers during the nursery season is confirmed, especially in smaller tributaries.

The results primarily indicate the occurrence of phenomena related to genetic drift, evidenced by a significant genetic distance between adult trout from Rega and Ina and the examined individuals from spawning areas and stocking material. However, it should be noted that the coefficient of variability values when comparing adult individuals and fry is inflated due to kinship relationships among juvenile individuals (Allendorf and Phelps 1981). This clearly illustrates why populations should not be compared based on juvenile individuals from narrow locations that are closely related (Hansen et al. 1997; Vera et al. 2010). Our findings also indicate that in most examined locations, the majority of individuals sampled by electrofishing had strictly originated from fish stocked in spring 2021.

The analysis revealed that adult sea trout from Ina and Rega Rivers clustered with resident brown trout from northern breeding lines rather than southern lines, which is logical and has already been pointed out (Bernaś and Wąs-Barcz 2020). However, considering gene flow between ecotypes, this information is crucial from a management perspective because it indicates from which breeding stocks stocking material should be utilized if we consider stocking with resident fish. This should be a priority that will support the preservation of biodiversity in populations associated with the lower Oder catchment area. Finally, considering the influence of stocking with anadromous Rega fish, it seems that stocking has the most homogenizing effect on the population. It is easy to imagine such a phenomenon. The annual distribution of thousands of closely related individuals into most tributaries may result in reduced polymorphism in the population and loss of genetic structure. However, the question arises whether serious changes in the population may have occurred long ago, resulting in, for example, inbreeding depression. The answer to this question may be the significant gene flow between individuals from natural spawning and those from stocking, and lower homing between stocked fish, which paradoxically could stabilize intrapopulation variability (Keefer and Caudill 2014).

## Conclusions

The results obtained in this study indicate several facts important for managing *Salmo trutta* populations in the Oder estuary basin. The first issue is the high proportion of individuals of stocking origin, which confirms the hypothesis of their possible dominance in areas where stocking is carried out during the nursery season. The results also indicate a lack of genetic differentiation between the sea trout populations from the Ina and Rega Rivers, showing that they cluster with the northern resident breeding stocks.

## Supplementary Information

Below is the link to the electronic supplementary material.

Supplementary file1 (XLSX 73 KB)

## Data Availability

Data are available upon request.
